# A Comparative Genomic Analysis Provides Novel Insights Into the Ecological Success of the Monophasic *Salmonella* Serovar 4,[5],12:i:-

**DOI:** 10.3389/fmicb.2018.00715

**Published:** 2018-04-17

**Authors:** Eleonora Mastrorilli, Daniele Pietrucci, Lisa Barco, Serena Ammendola, Sara Petrin, Alessandra Longo, Claudio Mantovani, Andrea Battistoni, Antonia Ricci, Alessandro Desideri, Carmen Losasso

**Affiliations:** ^1^Department of Food Safety, National Reference Center for Salmonella, Istituto Zooprofilattico Sperimentale delle Venezie, Legnaro, Italy; ^2^Department of Biology, University of Rome Tor Vergata, Rome, Italy; ^3^Science Communication Laboratory, Istituto Zooprofilattico Sperimentale delle Venezie, Legnaro, Italy

**Keywords:** *Salmonella*, tolerance to heavy metals, toxin-antitoxin, antimicrobial resistance, swine foodchain

## Abstract

Over the past decades, *Salmonella* 4,[5],12:i:- has rapidly emerged and it is isolated with high frequency in the swine food chain. Although many studies have documented the epidemiological success of this serovar, few investigations have tried to explain this phenomenon from a genetic perspective. Here a comparative whole-genome analysis of 50 epidemiologically unrelated *S*. 4,[5],12:i:-, isolated in Italy from 2010 to 2016 was performed, characterizing them in terms of genetic elements potentially conferring resistance, tolerance and persistence characteristics. Phylogenetic analyses indicated interesting distinctions among the investigated isolates. The most striking genetic trait characterizing the analyzed isolates is the widespread presence of heavy metals tolerance gene cassettes: most of the strains possess genes expected to confer resistance to copper and silver, whereas about half of the isolates also contain the mercury tolerance gene *merA*. A functional assay showed that these genes might be useful for preventing the toxic effects of metals, thus supporting the hypothesis that they can contribute to the success of *S*. 4,[5],12:i:- in farming environments. In addition, the analysis of the distribution of type II toxin-antitoxin families indicated that these elements are abundant in this serovar, suggesting that this is another factor that might favor its successful spread.

## Introduction

The epidemiology of *Salmonella* has been characterized by the recurring emergence of strains that periodically pose important threats to human health because of their pathogenicity, their persistence along the food and veterinary chain and their ability to spread widely. The past two decades has seen a rapid worldwide emergence of a new *Salmonella* serovar, namely monophasic variant of *S*. Typhimurium, which antigenic formula is 4,[5],12:i:-(Crayford et al., 2014). In spite of the similarity between this new serovar and *S*. Typhimurium, they show distinct epidemiological, genomic and phenotypic features. From an epidemiological point of view *S*. 4,[5],12:i:- is strongly associated to the swine food chain (Barco et al., 2015; EFSA, 2017), whereas *S*. Typhimurium displays a more heterogeneous source distribution being generally associated with cattle food chain, but also with other animal sources (EFSA, 2017). From a genomic point of view *S*. 4,[5],12:i:- variability is more limited than the *S*. Typhimurium one (Guerra et al., 2000; Agasan et al., 2002; Dionisi et al., 2009; Soyer et al., 2009; Barco et al., 2015). From a phenotypic point of view the two serovars differ for the lack of expression of the second flagellar phase (Soyer et al., 2009). Moreover, a significant difference has been described among the two serovars in terms of phage type diversity and antimicrobial resistance profiles (Barco et al., 2012).

According to recently published data by the European Food Safety Authority, *S*. Enteritidis is still the serovar that is more frequently identified as responsible for human and veterinary infections in Europe (EFSA, 2016). However, *S*. Typhimurium and *S*. 4,[5],12:i:- are the most common serovars among both human and veterinary isolates in Italy, Belgium, France, Denmark, England, and Galles (Pires et al., 2010; Hopkins et al., 2012). Interestingly, in some of these countries *S*. 4,[5],12:i:- has overtaken *S*. Typhimurium and now ranks as the top serovar isolated from animals and foodstuffs. The first *S*. 4,[5],12:i:- was identified in the late 1980s from poultry in Portugal (Machado and Bernardo, 1990) and between 1998 and 2004 it was the fourth most common serovar in Spain (de la Torre et al., 2003).

This initial clone of *S*. 4,[5],12:i:-, which was associated with pigs and pork products (de la Torre et al., 2003), was identified as the “Spanish clone” and showed a ASSuT MDR (Ampicillin Streptomycin Sulphonamides Tetracycline Multy Drug Resistances) phenotype with additional resistance to chloramphenicol, gentamicin and trimethoprim mediated by non-conjugative plasmids carrying the *spv* locus (Guerra et al., 2001; García et al., 2011, 2014; Loftie-Eaton and Rawlings, 2012). Moreover, isolates belonging to the Spanish clone were ascribed to phage type U302, ST-19, and differed from *S*. Typhimurium LT2 by five major deletions distributed along the chromosome (Garaizar et al., 2002).

A second major clonal line (the “European clone”), spread in several countries across the EU, was characterized by chromosomally encoded resistance to ampicillin, streptomycin, sulphonamides, and tetracyclines (EFSA, 2016) (ASSuT) and lacked the typical *S*. Typhimurium virulence plasmid (pSLT) (Hauser et al., 2010; Hopkins et al., 2010; Lucarelli et al., 2012; García et al., 2014). This clone was ascribed mainly to phage-types DT193 and DT120 and ST-34 (Bugarel et al., 2012).

*S*. 4,[5],12:i:- is a monophasic serovar because of the lack of expression of phase-2 flagellar antigen (Hopkins et al., 2012). In *Salmonella*, the two flagellar phases are expressed alternatively and flagellar phase variation depends on a switch of the transcription of *fliC* and *fljB* genes, which encode phase-1 and phase-2 flagellar antigens, respectively (Hopkins et al., 2012). This phenomenon, called “phase variation,” is based on the activity of a DNA invertase, encoded by the *hin* gene, which enables the reversible inversion of the promoter of the *fljAB* operon. This genetic element allows transcription of both *fljB* and *fljA*, encoding the phase-2 antigen and a negative regulator of the *fliC* gene, responsible for expression of the phase-1 flagellar antigen, respectively (Barco et al., 2014). Conversely, when the promoter is oriented to inhibit the expression of both *fljA* and *fljB*, phase-1 flagellar antigens can be detected because of the lack of repression of *fliC* (Soyer et al., 2009; Bugarel et al., 2012). The lack of expression of phase-2 flagellar antigen in *S*. 4,[5],12:i:- is generally associated with the deletion in *fljB* coding gene; however, to a less frequent extent, other mechanisms can lead to the monophasic status such as mutations and deletions of *fljA* and *hin* (Soyer et al., 2009). Furthermore, the invertible promoter that controls the expression of *fljB* and *fliC* may become blocked in one position, thereby allowing only the expression of the *fliC* gene (Zamperini et al., 2007). Several authors (Soyer et al., 2009; Hopkins et al., 2010; Bugarel et al., 2012; Barco et al., 2014) described variants of *S*. 4,[5],12:i:- that unexpectedly conserved the *fljB* gene even though are characterized by an inconsistent serological detection of the second flagellar phase. Such isolates have been named “inconsistent” *S*. Typhimurium or “atypical” monophasic variants of *S*. Typhimurium (atypical MVST hereafter), and their monophasic status is related to secondary mechanisms different from the absence of the *fljB* gene. Analysis of the genomic deletions in the phase-2 flagellum locus responsible for the monophasic phenotype of circulating *S*. 4,[5],12:i:- strains suggests that multiple independent clones may be emerging worldwide. A remarkable amount of genotypic variations that occurred during the expansion of this serovar, including multiple deletion events involving the phase-2 flagellin locus, were recently documented (Petrovska et al., 2016).

Although many studies have documented the ecological success of *S*. 4,[5],12:i:- serovar, few investigations have been conducted to explain this phenomenon from a genetic perspective. One accredited hypothesis to justify the epidemic spreading of different clones of *S*. 4,[5],12:i:- is the high occurrence of genes encoding tolerance to toxic concentrations of heavy metals and resistance to antibiotics (Mourão et al., 2016; Petrovska et al., 2016). It is broadly assumed that the spreading of strains showing high resistance to heavy metals has been promoted by the extensive use of feed supplemented with large amounts of copper and zinc to favor animal growth and to limit the use of antimicrobials. This feature is raising considerable concern because, in principle, it may affect the ability of the immune system to control bacterial infections, since macrophages are able to poison intracellular bacteria in the phagosome with large amount of copper and zinc (Neyrolles et al., 2015). Thus, the presence of additional heavy metal resistance genes may further contribute to the natural ability of *S*. 4,[5],12:i:- to escape the metal-mediated antimicrobial response of human macrophages (Kapetanovic et al., 2016). Moreover, the recent finding that *Salmonella* motility is modulated by zinc availability (Ammendola et al., 2016) suggests that the ability to face environments characterized by variable metal contents may contribute to the control of flagella expression in monophasic strains.

An additional factor that could be involved in the success of this emergent serovar is the presence of toxin-antitoxin (TA) cassettes. The involvement of these elements in a wide range of biological functions including growth control, defense against phages and biofilm formation may contribute to the success of the isolates harboring these genes in terms of adaptability to changing environments (Di Cesare et al., 2016). Moreover, it has been well established that TA systems, especially type II TA systems, play a central role in persistence (Page and Peti, 2016).

To identify the combination of different factors that could explain the eco-physiological role of this emergent serovar, whole genome sequencing of 50 epidemiologically unrelated *S*. 4,[5],12:i:- isolates obtained in Italy from animals and foodstuff from 2010 to 2016 was performed. Sequences were evaluated for the presence of genes conferring tolerance to heavy metals and antimicrobials, toxin-antitoxin systems, and specific plasmid replicons. A functional assay was performed to test the impact of heavy metals tolerance genes on bacterial motility in presence of high concentrations of copper. The presented results contribute to clarify the success and diffusion of the *S*. 4,[5],12:i:- serovar and provide valuable implications in terms of public health.

## Materials and methods

### Sample selection and collection

**Figure 1:**
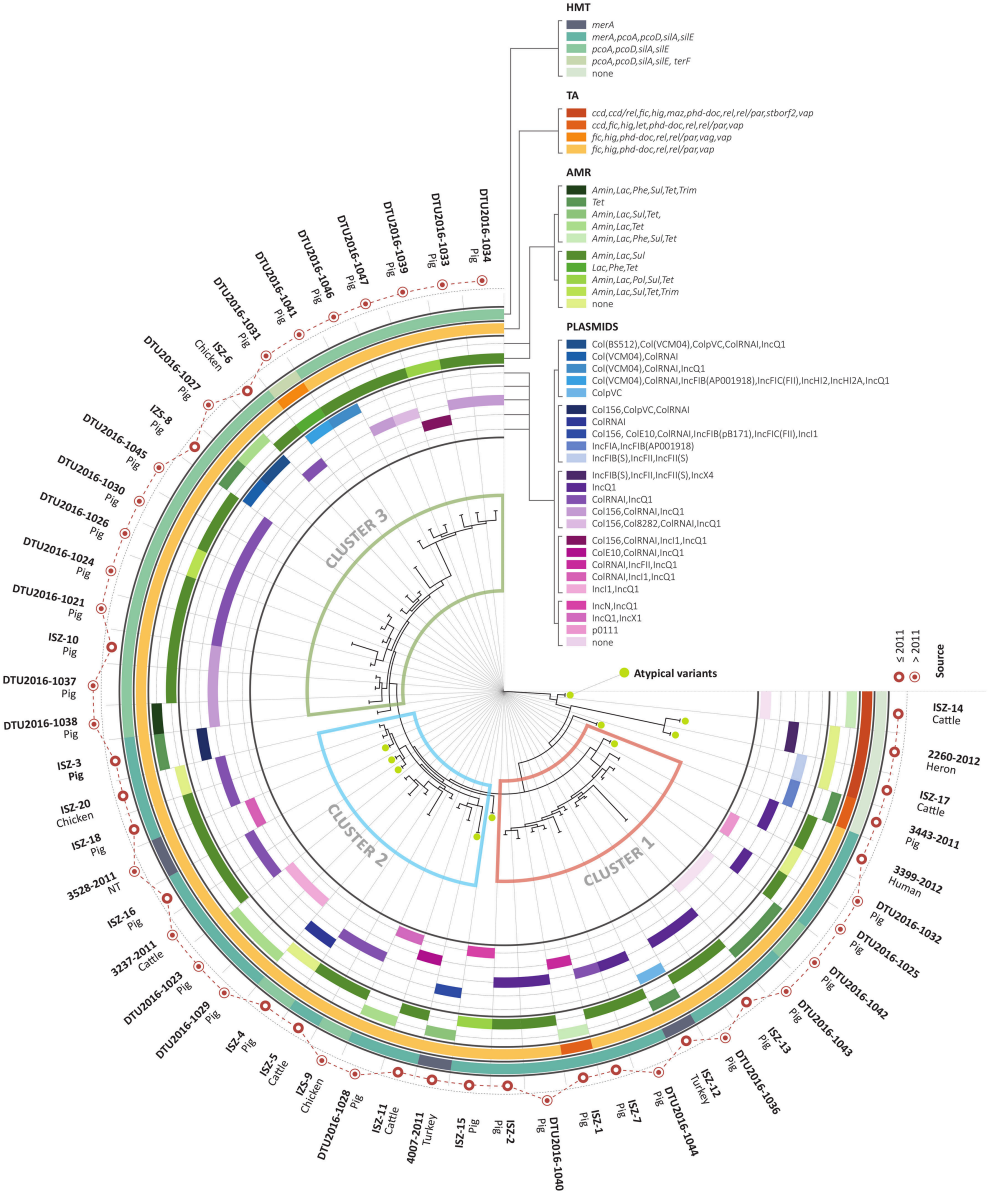
Summary infographic of strains. From outer to inner circle: strain name and source of origin; year of isolation (before/after 2011); HMT profile, color coded; TA profile, color coded; AMR profile, color coded (splitted into two circles for readability); replicon profile, color coded (splitted into five circles for readability); phylogenetic tree derived from core genome analysis. Strain clustering is highlighted (red: cluster 1; light-blue: cluster 2; green: cluster 3). Atypical MVST are highlighted with green circles. All 50 the analyzed strains and the two reference genomes (LN999997 and LT2) are included.

Fifty *S. enterica* serotype 4, [5],12:i:- isolates, also called monophasic variants of *Salmonella* Typhimurium (MVST), collected by the OIE Reference Laboratory for Salmonellosis (Istituto Zooprofilattico Sperimentale delle Venezie, Legnaro, Italy) and representative of the entire National situation from 2010 to 2016, were selected for this study. Complete information for the 50 investigated isolates is summarized in Figure 1, Supplemental Tables 1, 6. Most of the tested isolates were isolated in 2011 (*n* = 20) and 2015 (*n* = 25). Isolates were epidemiological unrelated, 10 of them were identified as atypical MVST, meaning that they phenotypically behave as MVST, even though the *fljB* gene is conserved (Barco et al., 2014).

The sources of the *Salmonella* isolates were: 37 from swine, 5 from poultry, 5 from bovine, 1 from humans, 1 from wild birds (heron), and 1 not assigned (Table 1).

**Table 1:** Sample description.

**SOURCE ORIGIN**					
CATTLE	PIG	POULTRY	NA	HUMAN	HERON
5 (3)	37 (3)	5 (1)	1 (1)	1 (1)	1 (1)
**YEAR OF ISOLATION**					
2010	2011		2012		2015	
2	20 (8)		3 (2)		25	
***IN-SILICO* MLST**					
ST-19	ST-34			ST-99	
2 (2)	46 (6)			2 (2)	

*Samples description in term of source, year of isolation and in-silico MLST. Numbers in brackets refer to the number of atypical MVST in the group*.

All isolates were serotyped according to the White-Kaufmann-Le Minor scheme (Grimont and Weill, 2007) using the traditional slide agglutination method. The *S*. 4, [5],12:i:- isolates were also phage typed following protocol developed by Anderson et al. (1977); the results were interpreted according to the guidelines established by the International Federation for Enteric Phage Typing (IFEPT, Laboratory of Enteric Pathogens, Health Protection Agency, Colindale, London, UK).

All isolates were supplied with all available information in terms of: country and region of isolation, collection date, and isolation source.

### Multilocus variable-number tandem repeat analysis

MLVA was performed according to the protocol described by Lindstedt et al. (2007). The size measurements for each locus were estimated using a Genetic Analyzer 3130XL (Applied Biosystems, Life Technologies Corporation, Carlsbad, CA, USA). According to the nomenclature suggested by Larsson et al. (2013), the MLVA results were reported as a string of five numbers representing the variable number of tandem repeats (VNTRs) at the corresponding loci (STTR9–STTR5–STTR6–STTR10pl–STTR3), or as “–” in the case that a PCR product was not obtained for a locus.

### Library preparation

Genomic DNA (gDNA) was extracted using commercial column-based protocols (DNeasy Blood and Tissue kit, Qiagen, and Easy-DNATM gDNA Purification kit, Invitrogen) and purified gDNA was quantified with a Qubit 3.0 Fluorometer (Life Technologies). Libraries for sequencing were prepared using a Nextera XT DNA sample preparation kit (Illumina): briefly, gDNA was subjected to tagmentation, a step in which the DNA is enzymatically cleaved and then tagged with Illumina adapters. Cleaved gDNA was subjected to a second PCR step for indexing with a unique combination of i5 and i7 index primers. PCR products were then purified using Agencourt AMPure XP beads (Beckman Coulter Inc.) and manually normalized to a final molarity of 4 nM using Tris-HCl (10 mM). High-throughput sequencing was performed with MiSeq Reagent kit v3 resulting in 251 bp long paired-ends reads. All the full-length sequences have been deposited in ENA (http://www.ebi.ac.uk/ena; accession number PRJEB21283).

### Sequencing and pre-processing

All the sequencing data were subjected to quality assessments using FastQC v0.11.2 and to length and quality trimming using Trimmomatic v 0.32 (Bolger et al., 2014), according to the following criteria: Illumina adapter removal; removing bases from the head and tail of the sequence if their quality score was below 20; removing sequences if the average quality score in a sliding window of four bases fell below 20; discarding sequences with a final length <100 base pairs.

### Bioinformatic analysis

All trimmed reads belonging to the same sample were assembled using Velvet v1.2.10 (Zerbino and Birney, 2008) and VelvetOptimizer v2.2.4 with options–s 19 (lower hash value)–e 99 (higher hash value)–t 10–f–fastq—shortPaired. Each sample was subject to three assembly runs and the assembly showing the higher n50 was chosen for each sample. All reads were then mapped onto the assembled bacterial genomes to compute the coverage statistics and to verify the quality of the assembly. When a sample showed an apparently large number of assembled contigs other statistics were evaluated, including n50, expected genome size, coverage statistics as well as other information deriving from sample annotation. The sample was considered for subsequent analysis when these metrics fell in a range comparable to the other samples.

All samples were subject to *in-silico* serotyping using SeqSero (Zhang et al., 2015) and SISTR (Yoshida et al., 2016), starting from both raw reads and assembled data, to confirm the *in vitro* serotyping.

### Identification of flagellar genes

Assembled data were searched for flagellar genes (flj genes UniProtKB identifiers: *fljA*—Phase-1 flagellin repressor: A0A0F7JBE2; *fljB*—Phase-2 flagellin: Q549S3) using the tblastn algorithm with default parameters to confirm the molecular characterization data available for atypical MVST isolates (Barco et al., 2014).

### Identification of heavy metal tolerance genes

Assembled data were searched for heavy metal tolerance by direct blasting of the nucleotide sequences of eight genes involved in tolerance against copper, tellurite, arsenite, and silver (Mourão et al., 2014) (Supplemental Table 8) using the blastn algorithm with the following options: percentage of identity between query and subject sequence >80%, query coverage >60%, best e-value (which was considered < 1e-15).

### Identification of antibiotic resistance genes and plasmid replicons

Assembled data were searched for antibiotic resistance genes (ARGs), plasmid replicons and subjected to *in-silico* multi locus sequence typing using the Bacterial Analysis Pipeline (https://cge.cbs.dtu.dk/services/cge/; Thomsen et al., 2016) with the following options: %ID threshold = 80.00%; selected minimum length = 60% for ResFinder and default parameters for all the other tools. In order to further investigate the presence of the *Salmonella* virulence plasmid pSLT, associated to the IncFIB(S) replicon in our database, all samples were searched for the *spv*C marker gene [NCBI, accession number NC_003277.2 (27178.27903, complement)] both by direct blasting with the following options: %ID threshold = 95%; minimum coverage length = 60%, e-value < 1e-15 and by investigation of the annotated genomes. Finally, in order to resolve whether the IncFIB(S) replicon was to be considered indicative of the pSLT (NCBI Reference Sequence accession number: NC_003277.2) or pSLT-BT (GenBank accession number: FN432031.1) plasmid, that share the same replicon, the whole plasmid reference sequences were searched in the genomes.

### Identification of type II toxin-antitoxin families

Assembled data were searched for type II toxin-antitoxin modules by directly blasting the TA database (downloaded from http://202.120.12.135/TADB2/download.html in date 08/29/2016; Shao et al., 2011) using the megablast algorithm with the following options: percentage of identity between query and subject sequence >80%, query coverage >60%, best scoring e-value (which was considered < 1e-15). Database hit were retained if and only if both the toxin and the corresponding antitoxin were contemporarily found in the same region of the assembled genome. Moreover, toxin-antitoxin matches were filtered as follows.

- Removal of multiple matches in the same position. When a multiple match was found, the one deriving from a manually curated reference annotated gene was kept, and the hits with genes with an annotation derived by protein homology were discarded. Matches deriving from a reference annotated by protein homology were kept if and only if there was no corresponding hit with other genes in the database with a manually curated annotation
- Removal of multiple matches in nearby and partially overlapping positions (hits in the same region but with ≤10-bp shift).

Toxin- antitoxin annotation was recovered from the corresponding references (in NCBI or ENA), and the toxin-antitoxin family was extracted; the final profile of the toxin-antitoxin family was determined for each sample by removing the replicate imputed family independently of the position of the hit.

Co-localization of ARGs, HMTG, and TAs on plasmids was investigated. A genomic region of interest was considered to be plasmidic if the same contig was flagged as containing the plasmid replicon sequence using PlasmidFinder.

### Phylogenetic analysis

#### SNP based phylogeny

Assembled data, S. Typhimurium LT2 reference genome (ENA ref AE006468.2) and the most recent reference of S. 4,[5],12:i:- (ENA ref LN999997.1) were subjected to SNP calling and reference-based phylogeny tree building using CSI Phylogeny 1.4 (Kaas et al., 2014) with LN999997.1 as the reference genome and default parameters for SNP filtering and pruning.

#### Core genome phylogeny

Assembled data were subjected to functional annotation using Prokka (Prokka, 2014). To compare our samples with pre-existing genomes references ENA ref AE006468.2 and ENA ref LN999997.1 were also annotated. Annotated data from all strains and the two references were passed to Roary v 3.6.2 (Page et al., 2015) (with options: –e –n –r) to build the pan-genome, core-genome and accessory genome of the strains.

Multiple alignment of core genes was converted from multifasta to phylip format, and then RAxML v 7.2.8 (Stamatakis, 2006) was used to infer the maximum-likelihood core genome phylogeny with the parameters -f a -x 12345 -p 12345 -# autoMRE -m GTRGAMMA. Phylogenetic trees obtained using the SNP-based approach and core genome multi-alignment were both re-rooted on the LT2 reference and compared using TOPD v4.6 (Puigbò et al., 2007) with the split distance (Robinson and Foulds, 1981) and nodal distance (Steel and Penny, 1993), and tested to verify whether the similarity between the two trees was significantly different from random.

#### Conjugation assay

The transfer frequency of *mcr-1* gene was investigated by conjugation experiments with rifampicin- resistant *E*. coli J53 as the recipient strain. Donor and recipient strains were grown in LB broth for 24 h at 37°C. A 1:50 dilution was prepared for each strain, and bacterial cells were grown at 37°C to an OD_600_ 0.4. Next, 500 μl of the donor strain was added to 4.5 ml of the recipient strain, and the bacterial suspension was filtered using a 0.22 μm filter laid down on a pre-heated agar tryptose (AT) plate and incubated for 18–24 h at 37°C. The filter was washed with 10 ml of physiological saline and vortexed to completely resuspend the cells. The cellular suspension was centrifuged at 5,000 rpm for 10 min, and the pellet was resuspended in 1 ml of physiological saline after removing the supernatant. Serial dilutions were prepared, and 100 μl was plated in triplicate on Luria Bertani (LB) plates supplemented with colistin (0.7 mg/L) and rifampicin (50 mg/L) to select for transconjugants. The transfer frequencies were calculated as the number of transconjugants obtained per recipient. To screen for the presence of *mcr-1* in transconjugant colonies, PCR was performed according to the protocol described by Liu et al. (2016). To determine whether the transconjugants also harbored the *hipBA* module, a PCR was designed for screening. DNA was extracted by boiling at 99°C for 15 min. PCR primers were as follows: HipA F 5′ GAATTCATGAGCCGGAAACAGCAA 3′; HipA R 5′ AAGCTTTCATTTCTCAGCCAGGC 3′; HipB F 5′ GAATTCATGATCAATAATGATTACCCGTTAAA 3′; HipB R 5′ AAGCTTTTACCAATCCTCCTGTCGT 3′. PCR was performed in a final volume of 50 μl using 1X Buffer Taq Gold, 2 mM MgCl2, 200 μM, or 400 μM dNTPs for HipB and HipA, respectively, 0,4 mM each primer, and 2,5 U Taq Gold (Life Technologies). Thermal cycling consisted of 95°C for 5 min, followed by 30 cycles (95°C for 30 s, 55°C for 30 s, 72°C for 90 s) and a final step at 72°C for 10 min for HipA. For HipB, the annealing temperature was 50°C and the elongation time was 30 s.

#### Screenings for swimming motility

A subset of 15 *Salmonella* strains were grown in Luria Bertani medium (LB) at 37°C with aeration. Strains were selected for carrying copper tolerance cluster (9 strains) or not (6 strains). In particular, among strains carrying tolerance cluster, IZS-11, IZS-15, IZS-16, DTU2016-1032, DTU2016-1036, DTU2016-1040, and DTU2016-1044 harbored *mer*A, *pco*A, *pco*D, *sil*A, *sil*E genes, DTU2016-1034 harbored *pco*A, *pco*D, *sil*A, *sil*E genes, and DTU2016-1031 harbored *pco*A, *pco*D, *sil*A, *sil*E, *ter*F. Strains IZS-14, IZS-17, 2260-2012, 3443-2011 did not harbor any tolerance genes among those investigated in the present study while strains 4007–2011 and 3528–2011 harbored only *mer*A gene.

Overnight grown inocula were diluted 1:100 in fresh medium and incubated at 37°C with aeration. After 5 h, 4 μl of each strain were dropped on LB motility plates (agar concentration 0,4%), supplemented or not with CuSO_4_ 2 mM. The plates were incubated with the upper side facing up at 37°C overnight. The next day halos of swimming bacteria were screened and their diameters recorded. Copper was prepared as a 0.5 M stock solution by solubilising ultra-pure powder purchased from BDH Laboratory Supplies in ultra-pure water (Ammendola et al., 2016).

Although literature data indicate that copper sulfate is not bactericidal for *Salmonella* at the concentrations used in this work (Pontel et al., 2007; Petrovska et al., 2016), preliminary assays were carried out to verify possible bactericidal activity of copper sulfate on all the strains under investigation. To this aim, inocula of *Salmonella* strains, grown overnight in LB medium, were washed once in PBS, resuspended in PBS to a final concentration of 10^8^ cfu/ml and then serially diluted (1:10) in the same buffer. 5 μl of each dilution were spotted onto LB agar plates supplemented or not with CuSO_4_ 2 mM. After an overnight incubation at 37°C the number of colony forming units on both plates was compared to highlight possible copper-induced bacterial killing.

#### Multivariate analysis

All the information recovered from previous analyses was summarized in a table to be imported in R (Team, 2008) for statistical analysis. Each category (heavy metal resistance gene profile, antimicrobial resistance profile, toxin-antitoxin system profile, imputed plasmid) was subjected to descriptive analysis, and an extended table in which each categorical variable was replaced with its dummy variable submatrix (i.e., where each gene was flagged as 1 if present in a specific sample, and 0 otherwise) was created for subsequent analysis. Plasmids, HMTGs, AMRs, and TA profiles were tested for co-localization in the same genomic region and for the significance of their association using Fisher's exact test. Multivariate analysis [implemented in the *FactoMineR* package (Lê et al., 2008)] was used for exploratory data analysis of the extended table, taking into account the grouped structure of the variables. The dummy variables describing AMRs, plasmids, HMTGs, and TAs (1 for presence, 0 for absence) were treated as qualitative variables; metadata variables (collection date, isolation source and region) together with microbiological and molecular variables (i.e., serotype and flagellin gene presence) were treated as supplementary categorical variables. Multiple factor analysis (MFA), data dimensionality and the influence of variable groups on the position of the samples in multidimensional space was investigated; visualization of the related graphs was performed using the package *factoextra* (Kassambara and Mundt, 2017).

Multilevel multinomial logistic regression [implemented through generalized linear models in the *glmnet* package (Friedman et al., 2010)] was used to test whether a relationship existed between the profiles of the strains in terms of plasmids, HMTGs, AMRs, TAs, and their cluster position in the phylogenetic trees determined in the previous steps. To investigate each feature weight in predicting sample grouping, only samples belonging to the main clusters identified in the phylogeny analyses were retained. Multinomial logistic regression was subjected to automatic K-fold cross-validation (*K* = 10) to select the optimal value of the regularization parameter lambda, which determined the overall strength of the lasso penalty. Minimization of misclassification error was set as the criterion for 10-fold cross-validation optimization. The value lambda.min, which is the value of lambda that provides the minimum mean cross-validated error, was selected for the final model.

The gene presence-absence table generated by Roary was imported into R. Each of the annotated gene of the pan-genome was set as 1 in all samples where they could be annotated, and 0 otherwise. Only accessory genomes (i.e., genes that were found in <52 samples) were used as input for hierarchical clustering using the Manhattan distance and Ward agglomeration method to investigate possible sample clustering based on genes shared by only a subset of the cohort. Therefore, predicted genes that were typical of each cluster (i.e., that were present in all strains of the cluster but were absent from all other strains) were investigated for function and GO terms by consulting UniProtKB.

For swimming motility analysis, the strains were divided into two groups according to the presence or absence of the copper resistance cluster. Mean and standard deviation values of motility in LB medium were calculated from replicate samples (*n* = 3) of each strain. The distribution of the data was tested for normality using the Shapiro-Wilk test, and differences in motility between two groups of samples were assessed using the Mann-Whitney U test.

All methods were carried out in accordance with relevant guidelines and regulations.

## Results

### *Salmonella* 4,[5],12:i:- sample characterization

Isolates were selected because they belonged to the most common phage-types associated with this serovar (DT193, U311, DT120, U302 DT20A, DT32, DT7, DT7a, RDNC, U302) and displayed different Multilocus variable-number tandem repeat analysis (MLVA) profiles (Supplemental Table 1). The *in silico* multilocus sequence typing (MLST) profile of the studied strains displayed a scenario in which the majority of the isolates belonged to ST-34 (*n* = 46; Table 1, Supplemental Table 1).

### Bioinformatics analysis

The raw data showed an average of 968707.94 ± 471051.53 sequenced reads with an average GC% content of 51.6% [range (51–53%)]. Quality trimming removed on average 8.14 ± 3.71% of the strain sequences. The assembled data displayed an average of 171.58 contigs with an average n50 of 242497.7 and an average coverage depth of 68.47 ± 33.72. Complete details are summarized in Supplemental Table 2. Blast research of genes involved in the flagellar structure revealed hits for the *fljA* and *fljB* genes on the assembled genomes of all the isolates (*n* = 10) known to be atypical MVST.

### Genomic regions encoding tolerance to heavy metals and resistance to antimicrobials

**Figure 2:**
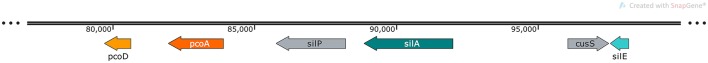
Location of pcoA, pcoD, silA, and silE in the identified genomic region. SilP and cusS are also represented in gray, since they were searched after functional annotation highlighted them as conserved in all samples carrying the pcoA-pcoD-silA-silE HMTG profile.

The heavy metal tolerance genes found were summarized into 5 HMTG profiles, as reported in Figure 1 and Table 2. Most strains (44/50) possess genes expected to confer resistance to both copper (*pcoA*-*pcoD*) and silver (*silA*-*silE*), with approximately half of them also exhibiting the mercury tolerance gene *merA*. In most of the strains, the *pcoA, pcoD, silA*, and *silE* genes, when present, were co-localized in the same genomic region (i.e., in the same contig), as reported in Figure 2, with *silE* preceding *silA* (5561-bp distance), *silA* preceding *pcoA* (4959-bp distance), and *pcoA* preceding *pcoD* (1321-bp distance).

**Table 2:** Sample description by genotype.

	**Number of samples (atypical MVST)**
**HMTG PROFILE**	
–	5 (4)
*merA*	3 (2)
*merA,pcoA,pcoD,silA,silE*	23 (4)
*pcoA,pcoD,silA,silE*	20 (0)
*pcoA,pcoD,silA,silE, terF*	1 (0)
**AMR PROFILE**	
*blaTEM-1B,strA,strB,sul2,tet(B)*	28 (4)
*tet(B)*	6 (1)
–	5 (2)
*aac(3)-IVa-like,aph(4)-Ia,blaTEM-1B,floR-like,strA,strB,sul2,tet(B),tet(C)*	2 (0)
*aac(3)-IVa-like,aph(4)-Ia,blaTEM-1B-like,blaTEM-1C-like,mcr-1-like,strA,strB,sul2,sul3,tet(A)-like,tet(B)*	1 (0)
*aadA1-like,aadA2,blaTEM-1B,cmlA1-like,dfrA12,strA,strB,sul2,sul3,tet(B)*	1 (0)
*aadA1,aadA2,blaTEM-1B,cmlA1-like,dfrA12,strA,strB,sul2,sul3,tet(A)*	1 (1)
*aadA1,blaTEM-1B,dfrA1,strA,strB,sul1,sul2,tet(A)-like*	1 (1)
*aph(3′)-Ia,blaTEM-1B,tet(B)*	1 (1)
*blaTEM-1B,dfrA14-like,strA-like,strB,sul2-like,tet(A),tet(B)*	1 (0)
*blaTEM-1B,floR-like,strA,strB,sul2-like,tet(B)*	1 (0)
*blaTEM-1B,strA-like,strB,sul2,tet(B)*	1 (0)
*blaTEM-1B,strA,strB,sul2*	1 (0)
*blaTEM-1B,strA,strB,sul2,tet(B),floR-like*	1 (0)
*blaTEM-1B,tet(B),floR-like*	1 (0)
**REPLICON PROFILE**	
Col(BS512),Col(VCM04),IncQ1,ColpVC,ColRNAI	1 (0)
Col(VCM04),ColRNAI	1 (0)
Col(VCM04),IncQ1,ColRNAI	1 (0)
Col(VCM04),IncQ1,ColRNAI,IncFIB(AP001918),IncFIC(FII),IncHI2A,IncHI2	1 (0)
ColpVC	1 (0)
ColpVC,ColRNAI,Col156	1 (0)
ColRNAI	1 (0)
ColRNAI,Col156,ColE10,IncFIC(FII),IncI1,IncFIB(pB171)	1 (1)
IncFIA,IncFIB(AP001918)	1 (1)
IncFIB(S),IncFII(S)	1 (0)
IncFIB(S),IncFII(S),IncFII	1 (1)
IncFIB(S),IncFII(S),IncX4,IncFII	1 (1)
IncQ1	8 (1)
IncQ1,ColRNAI	13 (2)
IncQ1,ColRNAI,Col156	6 (0)
IncQ1,ColRNAI,Col156,Col8282	1 (0)
IncQ1,ColRNAI,Col156,IncI1	1 (0)
IncQ1,ColRNAI,ColE10	1 (0)
IncQ1,ColRNAI,IncFIB(S),IncFII(S),IncFII	1 (1)
IncQ1,ColRNAI,IncFII	1 (0)
IncQ1,ColRNAI,IncI1	1 (1)
IncQ1,IncI1	2 (0)
IncQ1,IncN	1 (1)
IncQ1,IncX1	1 (0)
p0111	1 (0)
–	2 (0)
**TA PROFILE**	
*rel,fic,vap,hig,phd-doc,rel/par*	46 (6)
*rel,fic,vap,hig,phd-doc,rel/par,ccd,let*	2 (1)
*rel,fic,vap,hig,phd-doc,rel/par,ccd,maz,ccd/rel,stborf2*	3 (3)
*rel,fic,vap,hig,phd-doc,rel/par,vag, hip*	1 (0)

*Heavy metal tolerance (HMT) profile, replicon profile, antimicrobial resistance (AMR) profile, toxin-antitoxin (TA) profile and the corresponding number of strains in which they were found. Numbers in brackets refer to the number of atypical monophasic variants in the group. All 50 the analyzed strains and the two reference genomes (LN999997 and LT2) are included*.

No significant association was found between the presence of this multi-metal tolerance genomic region and the presence of other heavy metal tolerances genes in the same strain. However, Fisher's exact test confirmed a significant association between the presence of the profiles of multi-heavy metal tolerance genes (HMTGs) and the strains isolated after 2011 (p-value < 0.009). In detail, after 2011, more than 97% of the strains carried more than one HMTG, while ~70% showed this pattern before 2011 (Table 3); after 2011 more than 55% showed a HMTG profile containing either *merA* or *terF* in addition to the HMTG profile *pcoA, pcoD, silA, silE*, while only ~17% showed this pattern before 2011.

**Table 3:** Frequency of the heavy metal tolerance profile by year of strain isolation.

**HMTG**	**Year of isolation**	
	**≤2011**	**>2011**
none or *merA* only	7	1
*pcoA,pcoD,silA,silE*	12	11
*pcoA,pcoD,silA,silE* +1^*^	4	17

* *“+1” indicates the presence of another HMTG (merA or terF). All 50 the analyzed strains and the two reference genomes (LN999997 and LT2) are included*.

Moreover, as reported in Supplemental Table 3. The frequency analysis of annotated genomes revealed that the genomic region containing *merA* harbored other mercury tolerance genes such as *merC* (23 of 26 isolates) and *merP, merR*, and *merT* (21 of 26 isolates). Additionally, tetracycline resistance genes (*tetR, tetC*, and *tetA*) were found in the same region.

The *pcoA, pcoD, silA*, and *silE* region contains other copper and silver tolerance genes. In particular, *cusS* and *silP* co-occurred in 44 out of 44 strains (Figure 2), and *cusR, cusB, cusA, cusF, cusC*, and *pcoE* in 43 out of 44 strains. Furthermore, the arsenic tolerance genes *arsD, arsC, arsB* co-occurred in the same genomic portion for 42 out of 44 strains, and the cation tolerance genes *cutA* and *sugE* were found in the majority of strains (30 ≤ samples ≤ 40; Supplemental Table 3).

The same genomic region containing *terF* (found only in the strain DTU2016_1031) displayed the toxin-antitoxin cassette *hipB*-*hipA* and the tetracycline resistance gene *tetA* (Supplemental Table 3).

In most of the strains, the *pcoA, pcoD, silA*, and *silE* genes, when present, appeared to be co-localized in the same genomic region (i.e., contigs) containing the tetracycline resistance gene *tet*B. Forty samples showing *pco*A, *pco*D, *sil*A, *sil*E profile showed also the presence of *tet*B gene, while four samples showed *pco*A, *pco*D, *sil*A, *sil*E profile, and absence of *tet*B gene, respectively. Samples not showing *pco*A, *pco*D, *sil*A, *sil*E profile showed presence of the *tet*B gene in four samples, while four samples not showing *pco*A, *pco*D, *sil*A, *sil*E profile showed absence of the *tet*B gene. This association was confirmed by Fisher's exact test (*p*-value = 0.01365).

The acquired antimicrobial resistance gene profiles found in the panel of tested strains are summarized in Table 2. All but five samples showed at least one antimicrobial resistance gene, and most of them showed a complex pattern of multi-resistance. One strain (DTU2016_1031) was found to carry the colistin *mcr-1* resistance gene (%ID = 100%, query/HSP = 1,626/1,000).

In most strains, the sulfametoxazol resistance gene *sul2* and the streptomycin resistance genes *strA* and *strB*, when present, co-localized in the same genomic region (Fisher's exact test with *p*-value = 6.299e-11), which also contained the plasmid replicon IncQ1. Samples carrying the IncQ1 replicon showed such antimicrobial resistance (AMR) gene profile in 39 samples, while one sample carrying the IncQ1 replicon did not show such AMR gene profile. Samples not carrying the IncQ1 replicon did not show such AMR gene profile in 12 samples, while no sample did not carry the IncQ1 replicon nor such AMR gene profile.

### Replicon profile

The *in silico* plasmid identification revealed the presence of 22 different plasmid replicon types in the analyzed strains, whose profile are summarized in Table 2. Only two strains (DTU2016_1042 and DTU2016_1043) showed no hit in the plasmid replicon database; all other strains showed from one up to seven different predicted replicon types each, with most of the strains containing from one to three different replicon types. Plasmid replicon IncQ1 was the most common replicon type among the investigated strains being shared by 34 over 50 isolates, followed by replicon ColRNAI that was present in 27 over 50 isolates. All isolates displaying ST-34 lacked the IncFIB(S) replicon, which is a proxy of the *Salmonella* virulence plasmid pSLT in the used database (Kingsley et al., 2009; Carattoli et al., 2014; Silva et al., 2017), whereas isolates ISZ-14 (ST-19), ISZ-17 (ST-99), and 2260-2012 (ST-99), all classified as atypical MVST, showed a replicon profile containing IncFIB(S). Moreover, when the whole genome of these isolates was matched toward pSLT whole sequence, pSLT was retrieved with 100% of identity and 99% of reference coverage. These results are further supported by the finding that the *spvC* gene [which is considered to be a marker of the presence of pSLT (Herrero et al., 2009)] was present only in these three isolates.

### Genomic regions encoding type II toxin-antitoxin cassettes

The identified type II toxin-antitoxin cassette profiles are summarized in Table 2. All strains shared the putative presence of type II TA cassettes belonging to the *rel, fic, vap, hig, phd*-*doc*, and *par* family, while only a few strains showed additional TA modules belonging to the *ccd, let, maz, stborf*, *vag*, or *hip* family. In particular, TA modules belonging to the *ccd* family were identified in only five strains (3443-2011, ISZ-14, 2260/2012, ISZ-17, ISZ-1). For two of them (2260-2012, ISZ-17), the *ccd* TA box appeared to be co-localized on the same genomic region (i.e., the same contig) containing the replicon of the IncFIB(S) plasmid, suggesting that it may be carried by the plasmid. Complete details are summarized in Supplemental Table 9. No significant difference was found in total amount of TA modules (either divided by Type II family or cumulative) between samples isolated before/after 2011.

Interestingly in sample DTU2016_1031, the TA module *hip*A*/hip*B co-localized with *mcr-1* colistin resistance gene in a genetic region presumably of plasmidic origin, as supported by the concurrent presence of the IncHI2a plasmid replicon and many genes encoding conjugation abilities (e.g., bacterial conjugation TrbI-like protein, plasmid partition protein A, plasmid segregation protein ParM, type-F conjugative transfer system pilin assembly protein). In order to investigate the co-localization we found in the strain DTU2016_1031 of *mcr-1* and *hip*A*/hip*B genes on a plasmid and its relative transfer frequency, conjugation experiments with *E. coli* strain J53 were performed. The results demonstrated the plasmidic origin of *mcr-1* gene and a transfer frequency of 6 ^*^ 10-5 cells per recipient together with the *hip*A*/hip*B cassette.

### Phylogenetic analysis

**Figure 3:**
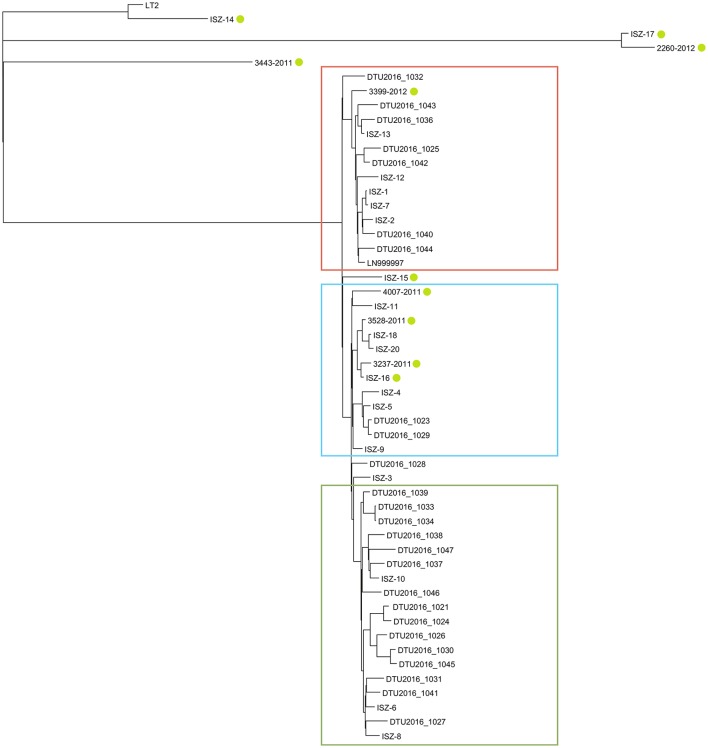
Phylogenetic tree obtained from SNP based phylogeny when LN999997 is used as reference strain; LT2 set as root; green circles highlight atypical MVST. Red: cluster 1; light-blue: cluster 2; green: cluster 3.

Single Nucleotide polymorphism (SNP) based phylogeny using LN999997.1 as a reference genome, which is a representative multidrug European clone(Petrovska et al., 2016) revealed 2102 high-quality shared SNP positions between each strain and this reference genome. The SNP distance between all strains ranged from 1 to 927 SNPs, generating the cladogram displayed in Figure 3 when *S*. Typhimurium LT2 (the reference) was used to re-root the tree.

As shown in Figure 3, three main clusters were identified. Cluster 1 (highlighted in red) comprises the *S*. 4,[5],12:i:- reference (ENA ref LN999997.1) and 13 strains, one of which was atypical MVST; the within maximum SNP distance among all strains in the cluster was 65 SNPs. Cluster 2 (highlighted in light blue) comprised 12 strains, four of which were atypical MVST; the within maximum SNP distance among all samples in the cluster was 60 SNPs. Cluster 3 (highlighted in green) comprised 18 strains, none of which was known to be atypical; this cluster included the only strain carrying the *mcr-1* resistance gene; the within maximum SNP distance among all samples in the cluster was 73 SNPs. If left unrooted, the tree highlighted another cluster grouping together all samples that were very distantly related from all other samples (847 SNPs), namely, 3443/2011, ISZ-14, ISZ-17, and 2260/2012, which were atypical MVST, and the LT2 reference (data not shown).

**Figure 4:**
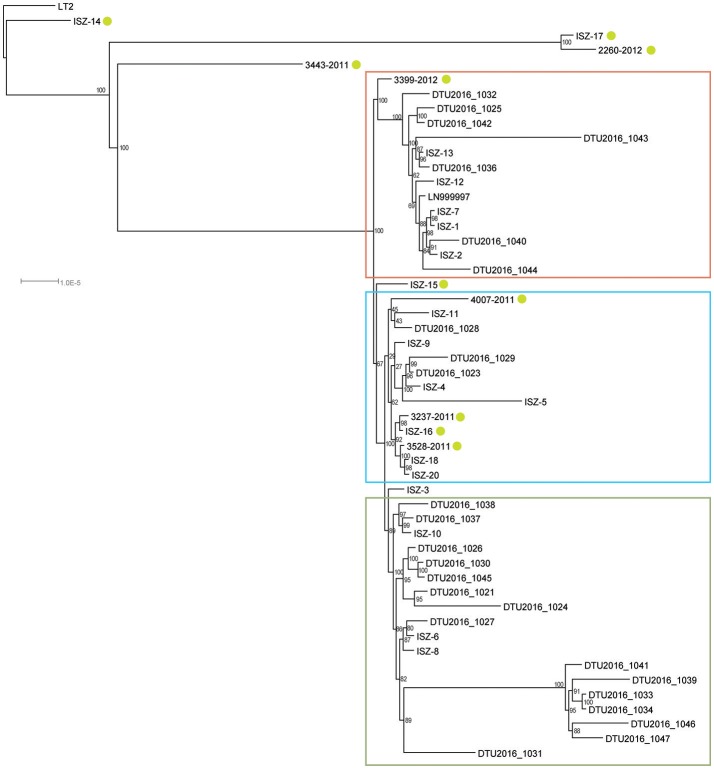
Phylogenetic tree obtained from core genome alignment phylogeny; LT2 set as root; green circles highlight atypical MVST. Red: cluster 1; light-blue: cluster 2; green: cluster 3.

Automatic annotation of assembled genomes identified an average of 4664.76 ± 77.86 CDS [range: (4552:5022)], an average of 75.53 ± 4.39 tRNA [range (67:87)] and a mode of three repeated regions per strain (Supplemental Table 2). The pan-genome calculation starting from the annotated files identified a core genome of 3983 genes (genes found in 99% ≤ strains ≤ 100%), 216 soft core genes (95% ≤ strains < 99%), and 593 shell genes (15% ≤ strains < 95%) over a pan-genome of 6992 genes. The multi-FASTA alignment of all the core genes consisted of 3799245 positions, 3799094 of which were determined. The best scoring maximum likelihood tree inferred from bootstrapping is displayed in Figure 4.

Three main clusters were identified with medium-to-high bootstrap value support. These clusters grouped the same strains identified by the SNP-based phylogeny, although sometimes with a different topology. If left unrooted, the cluster containing strains 3443-2011, ISZ-14, 2260-2012, and ISZ-17 and the LT2 reference appeared to be very distantly related to all other strains, in agreement with the SNP cluster assignment. The two phylogenetic trees achieved a split distance value of 0.326530612244898 and a nodal distance of 2.676077, indicating a good level of accordance between the two methods that was significantly different from random chance.

**Figure 5:**
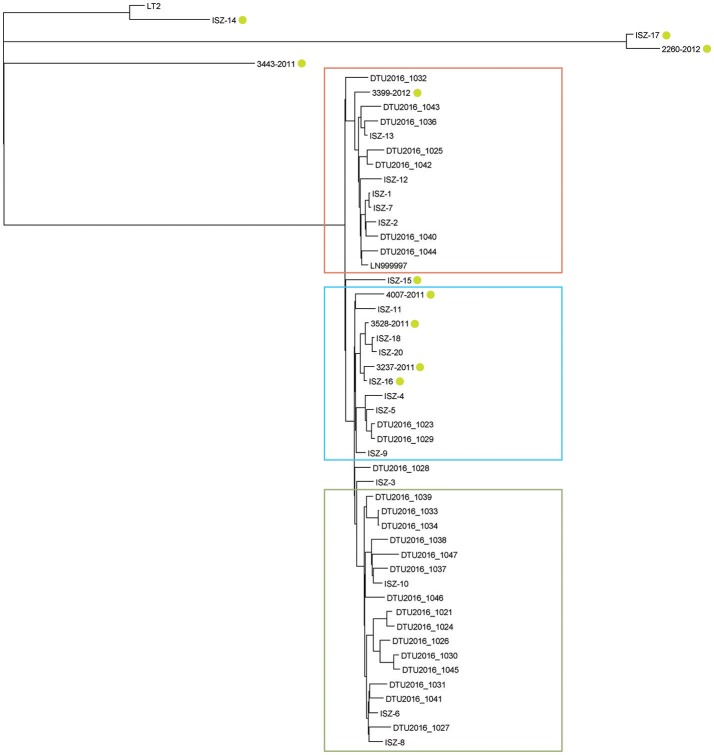
Hierarchical clustering with Manhattan distance and UPGMA over accessory genome presence/absence gene profile. All 50 the analyzed strains and the two reference genomes (LN999997 and LT2) are included.

Hierarchical clustering of the Manhattan distance computed over the accessory genome presence/absence (Supplemental Table 4) gene profile is depicted in Figure 5. An average of 645.7115 ± 77.32218 genes per strain was found in the accessory genome, with strain DTU2016_1031 showing the highest number of non-core genes (1,000) and ISZ-4 showing the lowest one (533). Again, strains 3443-2011, ISZ-14, 2260-2012, and 17 clustered with the LT2 reference distantly from all other strains. This cluster was characterized by 1194 accessory genes, 267 of which were common to all five strains. Although with some discrepancies, accessory genome hierarchical clustering showed that the strains tended to group depending on their clustering, as defined by the SNP-based or core genome-based phylogeny. Accessory genes that were typical of each cluster (i.e., that were present in all strains of each cluster, but were absent from all other strains) were annotated with function and GO terms as reported in Supplemental Table 5. In detail, cluster 1 was characterized by 1 predicted gene, cluster 2 by 2 predicted genes, cluster 3 by 15 predicted genes and samples outside clusters by 25 genes that were unique to each of the clusters of strains. Enriched GO terms (associated with ≥1 predicted genes) for each of the clusters were as follows.

1. Outliers—GO molecular function: structural molecule activity [GO:0005198]; GO cellular component: bacterial-type flagellum filament [GO:0009420], extracellular region [GO:0005576], cytoplasm [GO:0005737]; GO biological process: bacterial-type flagellum-dependent cell motility [GO:0071973]
2. Cluster 1—(no enrichment because there is only 1 gene)
3. Cluster 2—GO biological process: DNA binding [GO:0003677], metal ion binding [GO:0046872], transcription factor activity, sequence-specific DNA binding [GO:0003700], transcription, DNA-templated [GO:0006351]
4. Cluster 3—GO molecular function: DNA binding [GO:0003677]

### Multivariate analysis

Multivariate data analysis of the complete table (Supplemental Table 6) summarizing AMRs, plasmids, HMTGs and TAs as dummy variables together with metadata (collection year, isolation source and geographic area) and molecular/microbiological data (flagellin genes and ST) was performed. MFA was used to incorporate the grouped nature of variables (AMR, HMTG, TA, plasmids). The scree plot obtained from MFA highlights that the first two dimensions explained a good percentage of the total variance (>40%, data not shown), justifying the visualization of the 2D plot results. Six components were retained in the final results to ensure that most of the total original variance was captured.

**Figure 6:**
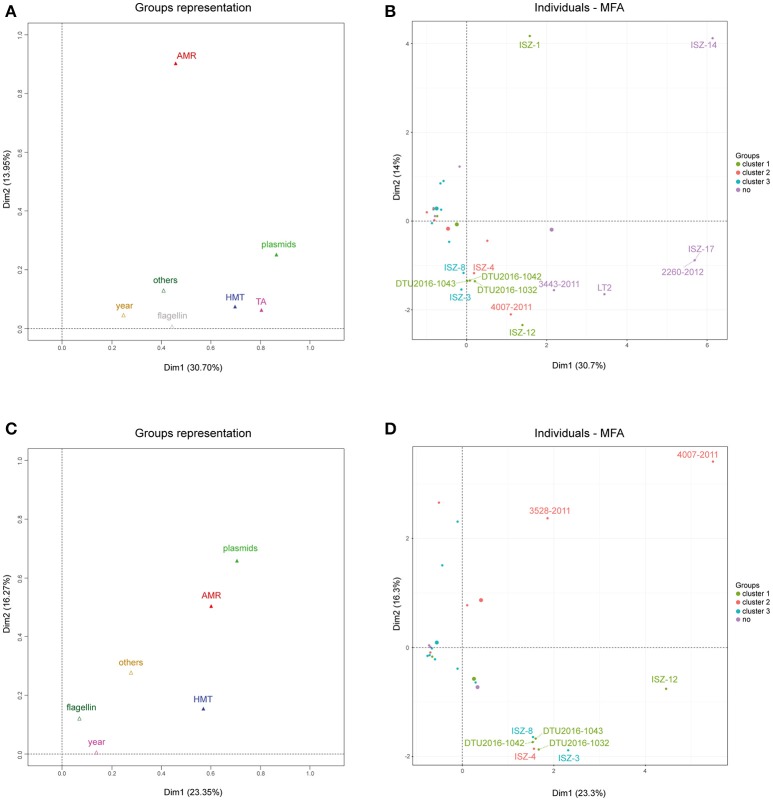
**(A)** Group representation on the first two dimensions for MFA on the whole dataset. **(B)** Individuals plot on the first two dimensions for MFA on the whole dataset. Color scales according to sample clustering in the phylogenetic analysis. **(C)** Group representation on the first two dimensions for MFA on the reduced dataset, after outliers removal. **(D)** Individuals plot on the first two dimensions for MFA on the reduced dataset, after outliers removal. Color scales according to sample clustering in the phylogenetic analysis. All 50 the analyzed strains and the two reference genomes (LN999997 and LT2) are included.

Figure 6A reports group representations for the first two dimensions. Toxin-antitoxin, heavy metal tolerance and replicon profile appeared to be the most influential factors in determining the positions of the strains in the first dimension, while antibiotic resistance profiles appeared to be the most influential factor in determining the position of the strain in the second dimension. The collection year and other metadata factors appeared to have a minor effect on the sample position in any of the two dimensions.

Figure 6B highlights the contribution of each strain to the construction of each of the first two dimensions and the position of the strain itself in the 2D plane.

The positions of strains 2260-2012, ISZ-17, and ISZ-14 were rightward according to their peculiar combination of TA profiles (*rel, fic, vap, hig, phd*-*doc, rel*/*par, ccd, maz, ccd*/*rel, stborf2*) and lack of HMTGs; moreover, strain ISZ-14 showed a unique AMR profile, while strains 2260-2012 and ISZ-17 showed no AMR gene. From this analysis, it appeared that almost all the influential strains (ISZ-14, 2260-2012, ISZ-17, LT2, 3443-2011) were actually those identified as outliers in the phylogenetic analysis. Therefore, to investigate finer differences in our cohort of strains, these outliers were excluded, and the analysis was re-run, discarding the variables responsible for their variance (Figures 6C,D) and showing the group representation and individual plots for this analysis.

After excluding the outliers, the percentage of variance captured by the first two axes declined by 7%. Moreover, the type II - TA modules were no longer useful in discriminating between strains (no variance shown in this subset of samples). The heavy metal tolerance profile was still an influential factor in determining the position of the strain in the first dimension, while the antibiotic resistance profile and plasmids appeared to be the influential factor in determining the position of the strain in both the first and second dimension. Strain 4007-2011, which has an extreme position in comparison to all other strains, appeared to be the most important contributor to construction of the axes, showing a unique profile both in terms of AMR (*tetB, floR*) and in the plasmid replicon profile. Strains ISZ-3, ISZ-4, ISZ-8, DTU2016_1032, DTU2016_1042, and DTU2016_1043 clustered together in the second quadrant. These strains had the following features:

- A diverse profile in terms of plasmid composition;
- No AMR or *tetB* gene only;
- A common TA profile;
- A HMTG profile with *pcoA, pcoD, silA*, and *silE*, with ISZ-3, DTU2016_1042 and DTU2016_1043 also showing *merA*.

A multinomial logistic regression was applied to investigate important variable for predicting strain clustering. This analysis was restricted to strains belonging to the three main clusters identified in the phylogenetic analysis (44 over 52 total strains), while seven of them were excluded (ISZ-3, ISZ-14, ISZ-15, ISZ-17, LT2, 3443-2011, 2260-2012). K-fold-cross-validation (*K* = 10) was used to optimize the regularization parameter lambda, resulting in lambda.min = 0.1086056 (value of lambda that provides the minimum mean cross-validated error). The final model, imposing lambda = lambda.min, identified 6 out of 25 variables as significant for determining the cluster label: *tetC* for AMR genes; ColRNAI, IncQ1, and IncI1 for plasmid replicons; *merA* for HM tolerance genes. Further details are supplied in Supplemental Table 7.

### Motility test

**Figure 7:**
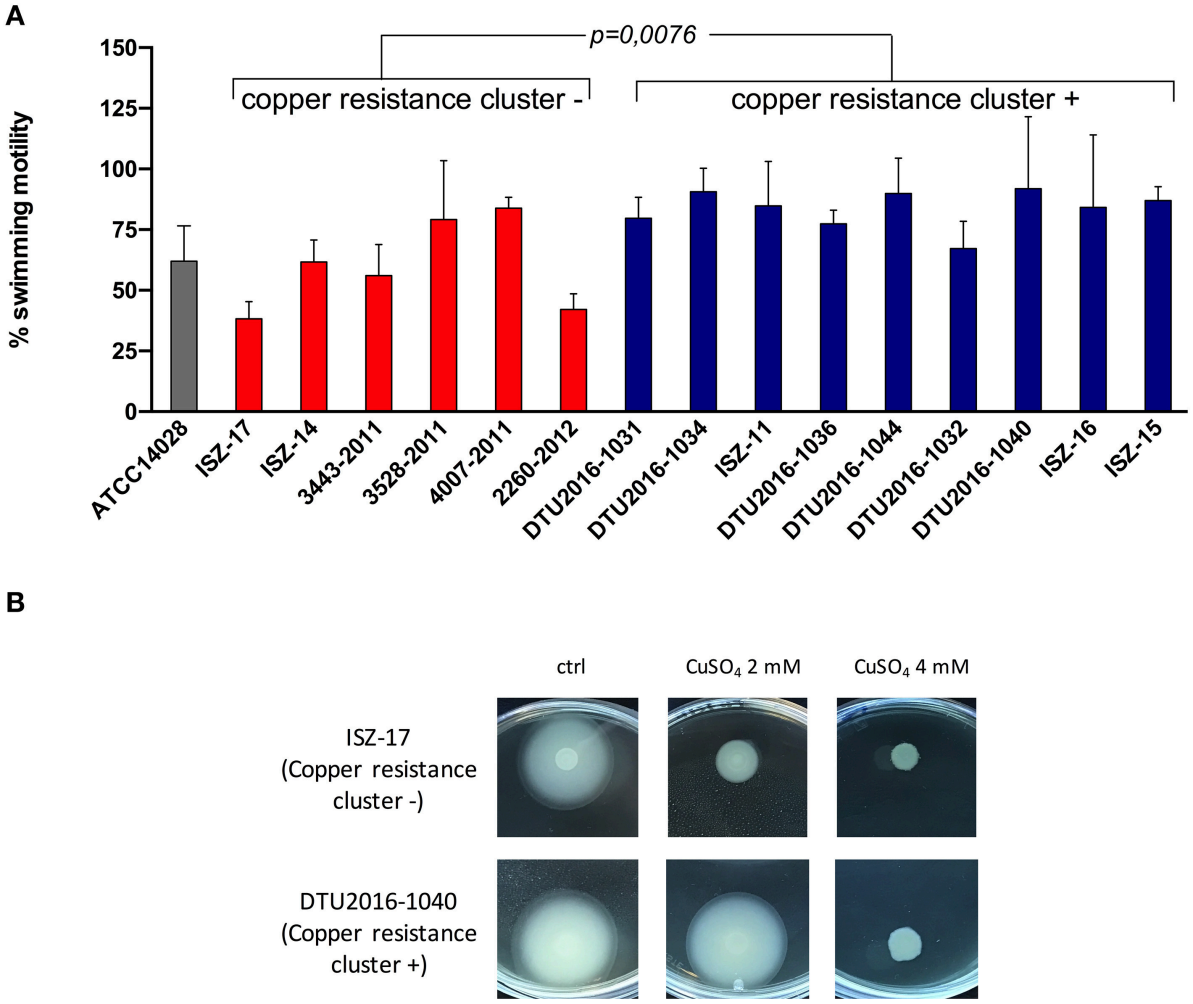
**(A)** Swimming motility in CuSO_4_ supplemented plates of *Salmonella* strains carrying the copper resistance cluster (blue bars) or not (red bars). Each percentage was calculated taking as 100% of motility the halo diameters in plates without CuSO_4_ supplementation. Statistical difference in motility between the two groups of samples was assessed by the Mann-Withney U test. **(B)** representative picture showing differences in swimming motility between strains carrying (ISZ-17) or lacking (DTU2016-1040) the copper resistance cluster. At high copper concentration (4 mM) motility is inhibited in both strains. The effect of copper on the motility of the biphasic strain ATCC 14018, which lacks the metal resistance gene cluster, in included for comparison.

To determine the effect of the *pco*A and *pco*D genes on *Salmonella* motility, 15 strains were tested using an agar motility test in LB medium supplemented or not with CuSO_4_ 2 mM, a concentration of metal that does not display bactericidal activity on *Salmonella* (Petrovska et al., 2016). The strains were divided into two groups: the first group consisted of samples for which copper resistance genes could not be annotated (*n* = 6); the second group comprised samples for which copper resistance genes could be annotated on the genome (*n* = 9). For comparison, we analyzed also the *S*. Typhimurium reference strain ATCC14028s, which lacks both *pco*A and *pco*D (Pontel et al., 2007), The halo diameter on LB plates was considered 100%, and the percentage of motility in the presence of copper was calculated for each strain (Figure 7). Strains carrying the copper resistance cluster were significantly less affected by the presence of the metal compared with the strains lacking those genes (Mann-Whitney U test, *p*-value = 0.0076).

## Discussion

In this study *S*. 4,[5],12:i:- was investigated with the aim of exploring genetic signatures possibly associated with its epidemiologic success. Genetic elements coding for tolerance to heavy metals, acquired antimicrobial resistance genes, type II toxin-antitoxin families and plasmid replicon types were studied by analyzing a number of 50 isolates belonging to different sources and collected in Italy from 2010 to 2016.

The results revealed the presence of common genetic features among the investigated isolates that might be involved in the widespread occurrence of such serovar in the swine food chain.

The main genetic trait shared by the investigated strains was represented by the presence of HMTGs encoding efflux systems involved in silver and copper tolerance, such as *pcoA, pcoD, silA*, and *silE*, as described by Mourão et al. for European and Spanish circulating clones of *S*. 4,[5],12:i:- (Mourão et al., 2015).

Tolerance to poisonous metals, mediated by different mechanisms, has been described as being on the rise among many bacterial pathogens (Losasso et al., 2014; Conficoni et al., 2016). This phenomenon has been documented by many authors, with a particular emphasis on pig husbandry (Medardus et al., 2014) and has been attributed to the use of the respective micronutrients in swine feed. Supplements such as copper and zinc, are added in swine and other livestock feed to promote animal growth and to increase feed efficiency. This practice has been rising since the ban of the use of antibiotics as growth promoters in animal feed, which has been effective in the EU since 2006 [Regulation 1831/2003/EC on additives for use in animal nutrition (1831/2003/EC, 2003)]. This use has been posing concerns among the scientific community regarding co-selection for antibiotic resistance in bacteria exposed to heavy metals (particularly copper and zinc but also iron, cobalt, manganese, and arsenic) that are used as growth promoters and for metaphylactic purposes in pig and poultry productions (Baker-Austin et al., 2006; Wales and Davies, 2015).

Unlike biocides (but with commonalities to antibiotic growth promoters), these micronutrients are used at inhibitory rather than lethal concentrations, potentially allowing tolerance phenomena to arise at a higher frequency in the farm environment than following biocide application (Wales and Davies, 2015). However, unlike antibiotic growth promoters, metals are very persistent in the environment (Wales and Davies, 2015). Moreover, due to agricultural practices as well as to other sources such as aquacultural and marine antifouling treatments, heavy metals may accumulate in soil, water, and sediments. Actually it has been estimated that livestock may be the major source of environmental contamination by zinc and copper (Nicholson et al., 2003; Wales and Davies, 2015). In the EU, high inclusion rates are permitted for young piglets which for inorganic zinc compounds may exceed basal requirements by thirty times (Baker-Austin et al., 2006; Norwegian Food Authority, 2014). Supplementation of ruminant feed with micronutrients is much less intensive than for pigs due to their high susceptibility to copper toxicity (Wales and Davies, 2015). The results of the present study indicate that *S*. 4,[5],12:i:- strains isolated after 2011 displayed a significantly higher prevalence of HMTGs than strains isolated before that date, suggesting that a time-dependent appearance of heavy metal tolerance profiles probably evolved under the selective pressure exerted by the rise in heavy metal concentrations in pig farming environments.

Flagella play an important role in the ability of *Salmonella* to colonize the gastrointestinal tract (Nicholson et al., 2003; Norwegian Food Authority, 2014; Ammendola et al., 2016) and some studies have indicated that flagellar gene expression is downregulated in presence of chelating agents or in strains that are unable to import zinc (Stecher et al., 2004; Ammendola et al., 2016). Zinc-dependent metabolic pathways are highly susceptible to interference by other transition metals that, when accumulating inside the cell, can compete with zinc for metal-binding sites in proteins (Ammendola et al., 2014). This scenario led us to speculate that *Salmonella* motility could be decreased in bacteria colonizing the gut of animals fed with copper enriched feed. As a matter of fact, we observed that *S*. 4,[5],12:i:- motility in agar plates containing 4 mM copper was completely abolished. Lower copper concentrations have variable effects on different *Salmonella* strains. We found that a 2 mM copper concentration inhibits motility of different *S*. 4,[5],12:i:- strains lacking the heavy metal resistance gene cluster, as well as that of the biphasic reference strain ATCC14028. A statistical comparison of the motility of *S*. 4,[5],12:i:- strains containing or lacking HMTGs in plates with 2 mM copper supported the hypothesis that the acquisition of this gene cluster promotes bacterial motility in environments enriched with this metal (Figure 7). Additional studies are required to consolidate this observation and to understand whether this feature may actually favor gut colonization in animals fed with copper-enriched feed. However, beyond the relevance of this specific observation, this experiment indicates that the acquisition of additional heavy metal resistance genes is useful for preventing the toxic effects of metals, thereby suggesting that this is a potential factor contributing to the success of this emerging *Salmonella* serovar.

The co-localization of silver and copper tolerance genes in the same genomic region, as well as mercury-encoding tolerance genes, suggests the possible acquisition of such determinants *via* the concerted actions of horizontal transfer of mobile elements. This hypothesis is also supported by the co-localization of genes involved in resistance to tetracycline (*tet* genes) in the same region harboring HMTGs and is coherent with the observation of Medardus et al. (2014) of a strong association between decreased susceptibility to heavy metals and antimicrobial resistance among *Salmonella* serovars isolated from swine, swine feed, and barn floors.

Genetic differences between MVST and atypical MVST isolates were detected beyond the well-known and described differences in the flagellin coding genes. Indeed, more than half of the atypical isolates displayed a reduced prevalence of HMTGs.

The frequency of antimicrobial resistance genotypes was high among *S*. 4,[5],12:i:- isolates with tetra-resistance causing the “ASSuT” phenotype, typical of the European clone, to be the predominant multiple drug resistance profile.

To investigate the potential mobilization of genetic elements, the panel of *Salmonella* isolates was examined for the presence of plasmid replicons, particularly those belonging to the major Inc family. The results revealed the presence of heterogeneous replicon contents in *S*. 4,[5],12:i:- isolates. The plasmid replicon IncQ1 was the most common among the isolates. The IncQ1 replicon is identical to the replicon of the broad-host-range RSF1010 plasmid of *E. coli*, containing the *strA, strB*, and *sul2* resistance genes. This element corresponds to the previously characterized IS26-Δ*repA, repC, sul2, strA*, and *strB*-IS26 transposon, in which part of the IncQ1 replicon has been mobilized together with the streptomycin and sulfonamide resistance genes (Miriagou et al., 2006). This mobile element is widely disseminated on plasmids and bacterial chromosomes (Izumiya et al., 2011) and was previously reported in an international collection of *S*. Typhimurium isolates originating mostly from humans but also from pigs (Helmuth et al., 1985). Plasmids of the IncQ family are highly mobilizable, stably maintained and transferred among a wide range of bacteria isolated from distinct environments (Smalla et al., 2000), thus suggesting their role in acquiring and transferring genetic material as well as in the concurrent bacterial survival ability in adverse environments. However, the phenotypic and genotypic high diversity of IncQ plasmids has been demonstrated, even for plasmids isolated from the same source (Helmuth et al., 1985; Izumiya et al., 2011). This finding suggests that the replicon may have evolved from a common ancestral plasmid; however, it is unlikely that IncQ plasmids represent a genetic trait for such classification purposes. It remains to be determined whether the resistance genes to sulfamides and streptomycins are located on the chromosomal or plasmidic portion of the isolate genomes, as the former hypothesis was demonstrated by Hendriksen et al. (2015). Since the presence/absence of the *Salmonella* virulence plasmid pSLT differentiates the European and Spanish clones of MVST circulating in Europe, the results support that the investigated isolates mainly belong to the European clone, being ST-34 and lacking pSLT. Concerning the atypical MVST, pSLT is harbored only by three out of the ten investigated isolates.

The analysis of the investigated strains genomes revealed the spread of type II TA, mainly involved in persistence phenomena. All the type II TA families were identified in the whole isolates panel. Additional genes were found in a small selection of strains. Type II TA modules are involved in bacterial adaptability in response to adverse environmental conditions and contribute to the non-growing phenotype in response to stress, allowing bacteria to self-protect becoming less sensitive to harmful environments (Hayes, 2003). These modules are frequently found in bacterial pathogens, including *Salmonella*, and are thought to be related to pathogenicity in epidemic bacteria (Georgiades and Raoult, 2011; Lobato-Márquez et al., 2015). Type II TA operons are likely to be acquired by bacteria through horizontal gene transfer and then positively selected due to the benefit given to pathogens in favorable ecological settings (Ramisetty and Santhosh, 2015).

Widespread and virulent *Salmonella* serovars, such as *S*. 4,[5],12:i:-, frequently harbor toxin-antitoxin (TA) cassettes (Di Cesare et al., 2016), that are located both in the chromosomal portion of the genome and/or in incompatible plasmids carrying ARGs, promoting persistence phenotypes (Ghafourian et al., 2014) and plasmid stabilization via post-segregation killing (Smalla et al., 2000; Georgiades and Raoult, 2011). *S*. 4,[5],12:i:- represents one of the three most prevalent serovars (EFSA, 2016), suggesting that the widespread occurrence of many type II TA families among the genome, as demonstrated in the present study, may represent a selective advantage supporting its successful spread.

In addition, the finding that the *mcr-1* gene co-localized with the *hip*A/*hip*B TA cassette only in the colistin-resistant *Salmonella* strain, together with the confirmation by the conjugation assay of the plasmidic origin of both *mcr-1* and *hip*A/*hip*B, suggests a possible active role for type II TA in enhancing plasmid stability and consequently the spread of *mcr-1*.

Both the SNP-based and the core genome-based phylogenetic analyses of the investigated isolates concordantly indicated a distinction among them based on the above-described genetic traits. Specific genetic signatures might be predicted for each of the identified clusters by accessory genome investigation; remarkably, metal ion binding genes could be identified as distinguishing cluster 2 from the others.

Atypical isolates spread among two of the three identified clusters and represented 100% of the isolates identified outside the clusters, close to the LT2 reference, lacking HMTGs, harboring pSLT and displaying a unique TA profile including additional TA modules (belonging to the *maz, ccd/rel* family plus *stborf*) compared to all the other analyzed strains.

Both phylogenetic trees highlighted the presence of a clade including isolates that were closely related the reference genome of *S*. 4,[5],12:i:- (LN999997), sharing a minimum genomic signature and including the most frequent type II TA families in the investigated panel (*rel, fic, vap, hig, phd-doc, rel/par*), *mer*A and *tet* genes. Moreover, the first and second clusters comprehensively collect all strains showing the presence of the mercury tolerance gene *merA* (either alone or in combination with *pcoA, pcoD, silA*, and *silE*), while all the strains considered to be outside any cluster lack all of the searched HMT genes. In addition, multinomial logistic regression supported the significance of a few genetic features, belonging to HMTGs, AMRG and the replicon profile, in discriminating the investigated strains according to the highlighted clusters. The multiple factorial analysis (MFA) obtained by including all the investigated genomic features confirmed that the type II TA families, ARGs, heavy metal tolerance genes and plasmid replicons concurrently explained the variance among the investigated isolates. However, based on the analysis excluding the outlier strains, a different picture was drawn in which the roles of the TA cassettes were lost and the ARGs and replicon types maintained their strength in explaining the observed variance, together with HMTGs, thus confirming the role of such genetic elements in characterizing *S*. 4,[5],12:i:-.

## Additional information

All the full-length sequences have been deposited in ENA (http://www.ebi.ac.uk/ena; accession number PRJEB21283).

## Author contributions

LB, AR, AB, AD, and CL: Conceptualization; EM, DP, LB, SA, AB, AR, and CL: Methodology; EM and DP; Formal analysis; SP, AL, SA, and CL: Investigation; EM, SP, and AL: Data curation; EM, LB, AB, SA, AD, and CL: Writing—original draft; CM: Visualization; AR: Funding acquisition; AR and CL: Supervision.

### Conflict of interest statement

The authors declare that the research was conducted in the absence of any commercial or financial relationships that could be construed as a potential conflict of interest.
