# Supplementary material for: A Comparative Genomic Analysis Provides Novel Insights Into the Ecological Success of the Monophasic Salmonella Serovar 4,[5],12:i:-
Source: Front Microbiol. 2018 Apr 17;9:715. doi: 10.3389/fmicb.2018.00715 (PMC5913373; doi:10.3389/fmicb.2018.00715)
Supplement: Supplementary file 7 [file DataSheet7.docx]

**TITLE:** **A comparative genomic analysis provides novel insights into the ecological success of the monophasic *Salmonella* serovar 4,[5],12:i:-**

E. Mastrorilli, D. Pietrucci, L. Barco, S. Ammendola^,^, S. Petrin, A. Longo, C. Mantovani, A. Battistoni, A. Ricci, A. Desideri, C. Losasso

**Supplemental_Table_S7. pdf** Significantly nonzero coefficients of the multinomial logistic regression model of all presence-absence data versus the cluster label (1-3)

| PREDICTORS | Cluster1 | Cluster2 | Cluster3 |
| --- | --- | --- | --- |
| Intercept | -0.7727650 | 0.22645478 | 0.54631017 |
| tetC | 0.6288335 | -0.55240235 | -0.07643117 |
| ColRNAI | 0.5834115 | -1.33336778 | 0.74995624 |
| IncQ1 | -0.1487267 | -0.05600971 | 0.20473639 |
| IncI1 | 0.4397579 | -0.41186725 | -0.02789065 |
| merA | 0.9455275 | 1.23569834 | -2.18122587 |
