# Supplementary material for: A Comparative Genomic Analysis Provides Novel Insights Into the Ecological Success of the Monophasic Salmonella Serovar 4,[5],12:i:-
Source: Front Microbiol. 2018 Apr 17;9:715. doi: 10.3389/fmicb.2018.00715 (PMC5913373; doi:10.3389/fmicb.2018.00715)
Supplement: Supplementary file 8 [file DataSheet8.docx]

**TITLE:** **A comparative genomic analysis provides novel insights into the ecological success of the monophasic *Salmonella* serovar 4,[5],12:i:-**

E. Mastrorilli, D. Pietrucci, L. Barco, S. Ammendola^,^, S. Petrin, A. Longo, C. Mantovani, A. Battistoni, A. Ricci, A. Desideri, C. Losasso

**Supplemental_Table_S8. pdf** Heavy metal resistance genes. Accession Number, Version and genomic region. Derived from Mourão et al., 2014 ^59^.

| HMRG | ACCESSION | VERSION | REGION |
| --- | --- | --- | --- |
| *arsB* | [BX664015](https://www.ncbi.nlm.nih.gov/nuccore/BX664015) | BX664015.1 | 159729..161018 |
| *merA* | K03089 | K03089.1 | 1753..3447 |
| *pcoA* | [X83541](https://www.ncbi.nlm.nih.gov/nuccore/X83541) | X83541.1 | 148..2084 |
| *pcoD* | [X83541](https://www.ncbi.nlm.nih.gov/nuccore/X83541) | X83541.1 | 3405..4334 |
| *silA* | [AF067954](https://www.ncbi.nlm.nih.gov/nuccore/AF067954) | AF067954.1 | 7117..10263 |
| *silE* | [AF067954](https://www.ncbi.nlm.nih.gov/nuccore/AF067954) | AF067954.1 | 895..1536 |
| *tcrB* | [AY048044](https://www.ncbi.nlm.nih.gov/nuccore/AY048044) | AY048044.2 | 4747..6879 |
| *terF* | [BX664015](https://www.ncbi.nlm.nih.gov/nuccore/BX664015) | BX664015.1 | 79946..81187 |
